# Optimization of Experimental Settings for the Assessment of Reactive Oxygen Species Production by Human Blood

**DOI:** 10.1155/2019/7198484

**Published:** 2019-01-10

**Authors:** Tânia Soares, Daniela Rodrigues, Mafalda Sarraguça, Sílvia Rocha, José L. F. C. Lima, Daniela Ribeiro, Eduarda Fernandes, Marisa Freitas

**Affiliations:** LAQV, REQUIMTE, Laboratory of Applied Chemistry, Department of Chemical Sciences, Faculty of Pharmacy, University of Porto, 4050-313 Porto, Portugal

## Abstract

The purpose of an experimental design is to improve the productivity of experimentation. It is an efficient procedure for planning experiments, so the data obtained can be analyzed to yield a valid and objective conclusion. This approach has been used as an important tool in the optimization of different analytical approaches. A D-optimal experimental design was used here, for the first time, to optimize the experimental conditions for the detection of reactive oxygen species (ROS) produced by human blood from healthy donors, a biological matrix that better resembles the physiologic environment, following stimulation by a potent inflammatory mediator, phorbol-12-myristate-13-acetate (PMA). For that purpose, different fluorescent probes were used, as 2′,7′-dichlorodihydrofluorescein diacetate (DCFH-DA), 2-[6-(4′-amino)-phenoxy-3H-xanthen-3-on-9-yl] benzoic acid (APF), and 10-acetyl-3,7-dihydroxyphenoxazine (amplex red). The variables tested were the human blood dilution, and the fluorescent probe and PMA concentrations. The experiments were evaluated using the Response Surface Methodology and the method was validated using specific compounds. This model allowed the search for optimal conditions for a set of responses simultaneously, enabling, from a small number of experiments, the evaluation of the interaction between the variables under study. Moreover, a cellular model was implemented and optimized to detect the production of ROS using a yet nonexplored matrix, which is human blood.

## 1. Introduction

The scientific research on reactive oxygen species (ROS), for a deeper insight into their biological functions and/or deleterious effects, still is a matter of intense research. Fluorescent probes have been mainly used to detect ROS in isolated cells, namely neutrophils [1, 2]. However, the isolation process itself often leads to artifactual cell activation, which represents an experimental confounder, being also expensive and time-consuming [3]. Moreover, in the detection of ROS, it is important to account the interaction of all blood components to resemble as closely as possible the *in vivo* physiologic state. In that sense, human blood is the most complex biological matrix that better resembles the physiological environment. There are just a few reports in literature about the detection of reactive species in human blood [3–5], but none of them described the experimental optimization of the method.

In this work, we use a D-optimal experimental design. This type of design is particularly useful when full factorial design cannot be applied due to experimental constrains, for example, when biological samples are used, as human blood. In a D-optimal design, the best subset of experiments is selected in order to maximize the determinant of the matrix X'X for a predetermined regression model. This means that the experimental runs chosen span the largest volume possible in the experimental region [6, 7]. Despite the usefulness of the D-optimal experimental design, this method is not usually applied to biologic matrices, being used here, for the first time, to optimize the experimental conditions for the *in vitro* detection of ROS produced by human blood cells, from healthy donors, following stimulation by a potent inflammatory mediator, phorbol-12-myristate-13-acetate (PMA), using different fluorescent probes, 2′,7′-dichlorodihydrofluorescein diacetate (DCFH-DA), 2-[6-(4′ -amino)-phenoxy-3H-xanthen-3-on-9-yl] benzoic acid (APF), and 10-acetyl-3,7-dihydroxyphenoxazine (amplex red). The variables tested were the human blood dilution, and the fluorescent probe and PMA concentrations. The experiments were evaluated using the Response Surface Methodology (RSM), and the method was validated using specific inhibitors of ROS production, for example, aminobenzoyl hydrazide (ABAH), diphenyleneiodonium chloride (DPI), N,N-dimethylurea (DMTU), and also a known antioxidant, the flavonoid luteolin.

## 2. Material and Methods

### 2.1. Chemicals

Dulbecco's phosphate buffer saline, without calcium chloride and magnesium (PBS), DCFH-DA, diphenyleneiodonium chloride (DPI), horseradish peroxidase (HRP), amplex red, catalase (from bovine liver), luteolin, and N,N-dimethylurea (DMTU), and phorbol-12-myristate-13-acetate (PMA) were obtained from Sigma-Aldrich Co. LLC (St. Louis, USA). 4-Aminobenzoyl hydrazide (ABAH) was obtained from Calbiochem (San Diego, CA, USA). APF was obtained from Invitrogen, Life Technologies Ltd. (Paisley, UK). The erythrocyte-lysing buffer (BD Pharm Lyse) was obtained from BD Biosciences (San Jose, CA, USA).

### 2.2. Blood Samples

All patient-related procedures and protocols were performed in accordance with Helsinki Declaration. Following informed consent, venous blood was collected, in the morning, from healthy human male and nonpregnant female volunteers aged 18–65 years. Experiments were performed within 30 min following blood collection.

### 2.3. Experimental Design

The optimization of the experimental conditions for the *in vitro* detection of ROS by DCFH-DA, amplex red, and APF was undertaken by using the RSM and an interaction D-optimal experimental design with 3 levels, two quantitative factors: probe and PMA concentrations, and a qualitative factor: blood dilution. The RSM methodology allows a deeper understanding of a product or process by optimizing and stablishing a robust experimental process.

All analyses were carried out at least six times, and the mean data obtained in the experiments was analyzed using the RSM so as to fit the model equation that related the response to the factors varied by the Modde software version 10.1.1 (Umetrics AB, Umeå, Sweden). In order to correlate the response variable to the independent variables, multiple linear regression was used to fit the coefficient of the model. The model goodness-of-fit was evaluated using analysis of variance (ANOVA) at the level of 95% of significance. For all models, the R^2^ and the Q^2^ values were calculated. R^2^ is an indication of the model fit, and Q^2^ shows an estimate of the future prediction precision. Q^2^ should be greater than 0.1 for a significant model and greater than 0.5 for a good model.

To validate the optimization, at least six experiments were conducted under the chosen optimal conditions to compare to the results obtained by the experimental design and, in this way, allowing to verify the predictability ability of the model.

### 2.4. Detection of ROS

Experimental optimization was conducted for each of the three probes under study. After this optimization, the method effectiveness was attested by using the following compounds: specific inhibitors of the enzymes responsible for the generation of reactive species as DPI (NADPH oxidase inhibitor) [8] and ABAH [myeloperoxidase (MPO) inhibitor] [9], the scavenger of hydrogen peroxide (H_2_O_2_), DMTU [10], and catalase, that catalyzes H_2_O_2_ decomposition into H_2_O [11].

The known antioxidant flavonoid luteolin [12] was also tested to validate the method. The antioxidant activity of luteolin has been associated with their ability to scavenge ROS, as anion radical superoxide (O_2_^·-^) and hypochlorous acid (HOCl) [13], to inhibit the prooxidant enzymes NADPH oxidase [14–16] and myeloperoxidase (MPO) [17], and to chelate transition metals involved in Fenton reaction [18]. All the concentrations cited in the following sections are final concentrations.

#### 2.4.1. DCFH-DA Assay

*(1) Experimental Optimization*. DCFH-DA is a nonpolar and nonfluorescent molecule that has the ability to diffuse through cell membranes into the cytoplasm where it is enzymatically cleaved by intracellular esterases to the polar nonfluorescent 2′,7′-dichlorodihydrofluorescein (DCFH). This molecule then becomes trapped inside the cell and is oxidized by ROS, producing the highly fluorescent 2′,7′ dichlorofluorescein (DCF) [19].

Whole blood (630 *μ*L), diluted in PBS (ratio of 1 : 5, 1 : 10, and 1 : 20), was placed in 24-well plates and incubated in a humidified atmosphere with 5% CO_2_ at 37°C, with 100 *μ*L of DCFH-DA (50, 100, and 200 *μ*M) during 30 minutes. Then, 20 *μ*L of PBS (same volume used for inhibitors) was added and incubated with the mixture for 15 minutes. In sequence, 50 *μ*L of PMA (100, 200, and 400 nM) was added. The fluorescence was measured at *λ*_excitation_ = 485 ± 20 nm and *λ*_emission_ = 528 ± 20 nm in a microplate reader (Cytation 3, Biotek, Vermont, USA). The D-optimal design consisted on a total of 16 experiments with 2 central points. Effects are expressed as the percentage of DCFH-DA oxidation, comparing with the blank (without PMA).

*(2) Effect of ROS Production Inhibitors*. Whole blood (diluted 1 : 20 in PBS) was placed in 24-well plates and incubated in a humidified atmosphere with 5% CO_2_ at 37°C, with DCFH-DA (120 *μ*M) during 30 minutes. Then, DPI (0-5 *μ*M), ABAH (0-2.5 mM), DMTU (0-60 mM), catalase (0-1300 U/mL), or luteolin (0-100 *μ*M) were added and incubated with the reaction mixture for 15 minutes. In sequence, PMA (120 nM) was added. The fluorescence was measured as previously described in the Experimental Optimization section (2.4.1. Effects are expressed as the percentage of inhibition of DCFH-DA oxidation, comparing with the control (with PMA).

#### 2.4.2. Amplex Red Assay

*(1) Experimental Optimization*. Amplex red is a highly specific and sensitive fluorogenic probe for the detection of extracellular H_2_O_2_. Amplex red is a colourless and nonfluorescent compound that, when oxidized by H_2_O_2_, in the presence of HRP, originates resofurin, which is a highly fluorescent product [20].

Whole blood (630 *μ*L), diluted in PBS (ratio of 1 : 5, 1 : 10, and 1 : 20), was placed in 24-well plates and incubated in a humidified atmosphere with 5% CO_2_ at 37°C, with 25 *μ*L of HRP (1 U/mL) and 25 *μ*L of amplex red (6.3, 12.5, and 25 *μ*M) during 10 minutes. Then, 20 *μ*L of PBS (same volume used for inhibitors) was added and incubated with the mixture for 15 minutes. In sequence, 50 *μ*L of PMA (25, 100, and 200 nM) was added. The fluorescence was measured at *λ*_excitation_ = 560 ± 20 nm and *λ*_emission_ = 585 ± 20 nm in a microplate reader (Cytation 3, Biotek, Vermont, USA). The D-optimal design consisted on a total of 17 experiments with 3 central points. Effects are expressed as the percentage of amplex red oxidation, comparing with the blank (without PMA).

*(2) Effect of ROS Production Inhibitors*. Whole blood (diluted 1 : 20 in PBS) was placed in 24-well plates and incubated in a humidified atmosphere with 5% CO_2_ at 37°C, with HRP (1 U/mL) and amplex red (10 *μ*M) during 10 minutes. Then, DPI (0-2.5 *μ*M), ABAH (0-500 *μ*M), catalase (0-1000 U/mL), DMTU (0-60 mM), or luteolin (0-100 *μ*M) were added and incubated with the reaction mixture for 15 minutes. In sequence, 150 nM of PMA was added. The fluorescence was measured as previously described in the Experimental Optimization section (2.4.2). Effects are expressed as the percentage of inhibition of amplex red oxidation, comparing with the control (with PMA).

#### 2.4.3. APF Assay

*(1) Experimental Optimization*. APF is a nonfluorescent derivative of fluorescein that originates fluorescein intracellularly, by *O*-dearylation, upon reaction with HOCl, hydroxyl radical (HO^·^) and peroxynitrite anion (ONOO^−^) leading to its characteristic fluorescence [21].

Whole blood (630 *μ*L), diluted in PBS (ratio of 1 : 5, 1 : 10, and 1 : 20), was placed in 24-well plates and incubated in a humidified atmosphere with 5% CO_2_ at 37°C, with 100 *μ*L of APF (1, 5, and 10 *μ*M) during 10 minutes. Then, 20 *μ*L of PBS was added and incubated with the mixture for 15 minutes. In sequence, 50 *μ*L of PMA (50, 200, and 400 nM) was added and incubated for 30 minutes.

The samples were subsequently transferred to conic tubes with 8 mL of an erythrocyte lysing solution (BD Pharm Lyse), according to the manufacturer specifications and incubated at room temperature, protected from light, for 15 minutes. Then, samples were centrifuged at 200 g for 5 minutes, followed by the removal of the supernatant. The pellet was resuspended in 300 *μ*L of PBS. The fluorescence was measured at *λ*_excitation_ = 485 ± 20 nm and *λ*_emission_ = 528 ± 20 nm in a microplate reader (Cytation 3, Biotek, Vermont, USA). The D-optimal design consisted on a total of 16 experiments with 2 central points. Effects are expressed as the percentage of APF oxidation, comparing with the blank (without PMA).

*(2) Effect of ROS Production Inhibitors*. Whole blood (diluted 1 : 20 in PBS) was placed in 24-well plates and incubated in a humidified atmosphere with 5% CO_2_ at 37°C, with APF (5.5 *μ*M) during 10 minutes. Then, DPI (0-5 *μ*M), ABAH (0-2.5 mM), DMTU (0-60 mM), or luteolin (0-100 *μ*M) were added and incubated with the reaction mixture for 15 minutes. In sequence, PMA (150 nM) was added and incubated for 30 minutes.

Then the samples were treated as mentioned in the Experimental Optimization section (2.4.3). Effects are expressed as the percentage of inhibition APF oxidation, comparing with the control (with PMA).

### 2.5. Statistical Analysis

The IC_50_ value (concentration that reduces the studied effect by 50%) was calculated using GraphPad Prism™ (version 7.0; GraphPad Software). Results are expressed as mean ± standard error of the mean (SEM) (from at least three individual experiments, performed in triplicate in each experiment).

## 3. Results

### 3.1. Optimization of Experimental Settings in the DCFH-DA Assay

The examination of the model regression coefficients (*p* < 0.05) showed that the qualitative factor (blood dilution) was not significant for the model and that the oxidation increases with the increase of DCFH-DA and PMA concentrations. The model for the percentage of DCFH-DA oxidation [equation (1)] fitted the experimental data with a R^2^ of 0.95 and a Q^2^ value of 0.88, demonstrating that a model with a good fit and good predictability ability was obtained.

(1) $$\begin{array}{c} Oxidation\text{ of }DCFH-DA\left(\%\right)=0.080+3.060X_{1}+0.150X_{2}–0.009{X_{1}}^{2}. \end{array}$$

**Figure 1:**
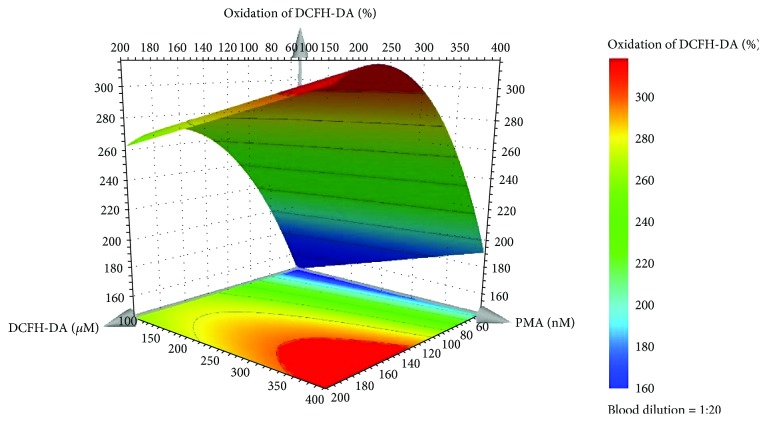
Response surface plot obtained for the percentage of oxidation of the DCFH-DA by the predictive model of the D-optimal design for the 1 : 20 blood dilution.

In equation (1), *X*_1_ is the DCFH-DA concentration (*μ*M) and *X*_2_ is the PMA concentration (nM). Taking into account that all tested dilutions of human blood originated a good range of oxidation percentage of the probe, we choose the 1 : 20 dilution in order to use less quantity of human blood in each assay, making the biologic sample more profitable. Equation (1) and the response surface plot (Figure 1) allow us to choose the percentage of oxidation of the probe that better fits our aims. In this case, to obtain a probe oxidation around 250% (indicative value to validate the Equation), the concentrations of DCFH-DA and PMA should be 120 *μ*M and 120 nM, respectively. The validation of the model was executed (*n* = 6), and the results showed that the percentage of oxidation of the probe was around the expected value (232 ± 45%). No significant difference (*p* < 0.05) was found between the validation experiments and those predicted by the model, confirming the good model prediction ability.

**Figure 2:**
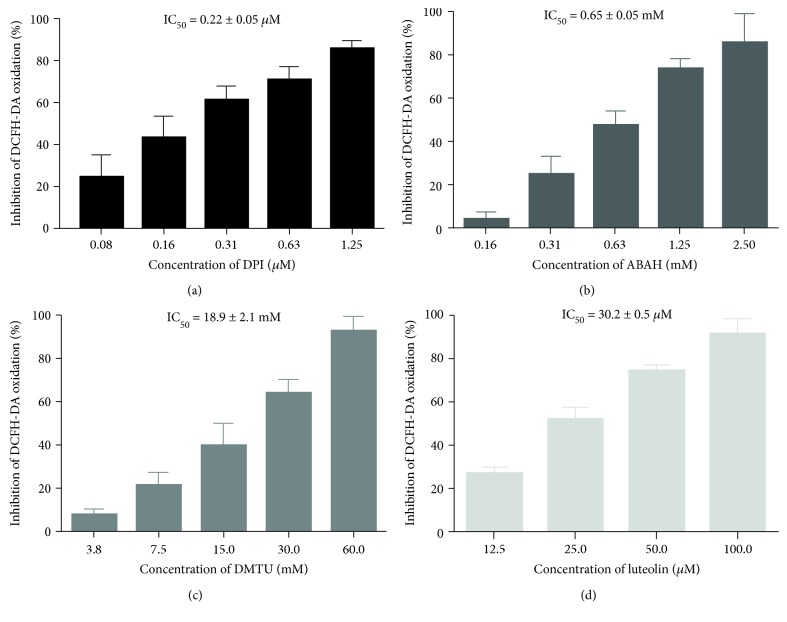
Inhibitory effects of DPI (0.08-1.25 *μ*M) (a), ABAH (0.16-2.50 mM) (b), DMTU (3.8-60 mM) (c), and luteolin (12.5-100 *μ*M) (d) on the oxidation of DCFH-DA, by whole blood-generated ROS, when stimulated by PMA. Values are given as mean ± SEM (*n* ≥ 3).

To better understand which reactive species are involved in the oxidation of DCFH-DA in this model, we used DPI (inhibit NADPH oxidase), catalase (catalyzes H_2_O_2_ decomposition into H_2_O), DMTU (scavenges H_2_O_2_), ABAH (inhibitor of MPO), and luteolin (a known antioxidant). As it can be seen in Figure 2, among the inhibitors used, only DPI, ABAH, and DMTU avoided the oxidation of DFCH-DA in a concentration-dependent manner. The IC_50_ values obtained for DPI was IC_50_ = 0.22 ± 0.05 *μ*M, for ABAH was 0.65 ± 0.05 mM, and for DMTU was IC_50_ = 18.9 ± 2.1 mM. Catalase increased the fluorescence value *per se*, suggesting a possible interference with the methodology. These results indicate that, using human blood as cellular model, DCFH-DA preferentially detects NADPH oxidase-derived ROS as H_2_O_2_ and HOCl. Luteolin originated an IC_50_ = 30.2 ± 0.5 *μ*M.

### 3.2. Optimization of Experimental Setting in the Amplex Red Assay

The examination of the model regression coefficients (*p* < 0.05) showed that the qualitative factor (blood dilution) was not significant for the model and that the oxidation increases with the increase of amplex red and PMA concentration. The model for the percentage of amplex red oxidation [equation (2)] fitted the experimental data with a R^2^ of 0.85 and a Q^2^ value of 0.67 demonstrating that a model with a good fit and good predictability ability was obtained.

(2) $$\begin{array}{c} Oxidation\text{ of }amplex\;red\left(\%\right)=1174.07–3.184X_{1}+1.093X_{2}. \end{array}$$

In equation (2), *X*_1_ is the amplex red concentration (*μ*M) and *X*_2_ is the PMA concentration (nM). Taking into account that all tested dilutions of human blood originated a good range of oxidation percentage of the probe, we choose the 1 : 20 dilution in order to use less quantity of human blood in each assay, making the biologic sample more profitable.

**Figure 3:**
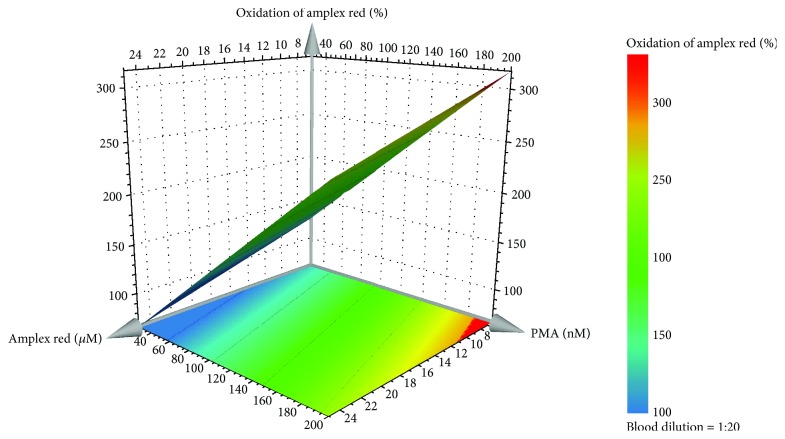
Response surface plot obtained for the percentage of oxidation of amplex red by the predictive model of the D-optimal design for the 1 : 20 blood dilution.

In this case, we also choose a probe oxidation around 250% as an indicative value to validate the Equation. According to equation (2) and as it can be seen in the response surface plot (Figure 3), to obtain a percentage of probe oxidation around 250%, a concentration of amplex red and PMA of 10 *μ*M and 150 nM, respectively, was chosen. The validation of the model was executed (*n* = 6), and the results showed that the percentage of oxidation of the probe was around the expected value (268 ± 18%).

No significant difference (*P* < 0.05) was found between the validation experiments and those predicted by the model, confirming the good model prediction ability.

**Figure 4:**
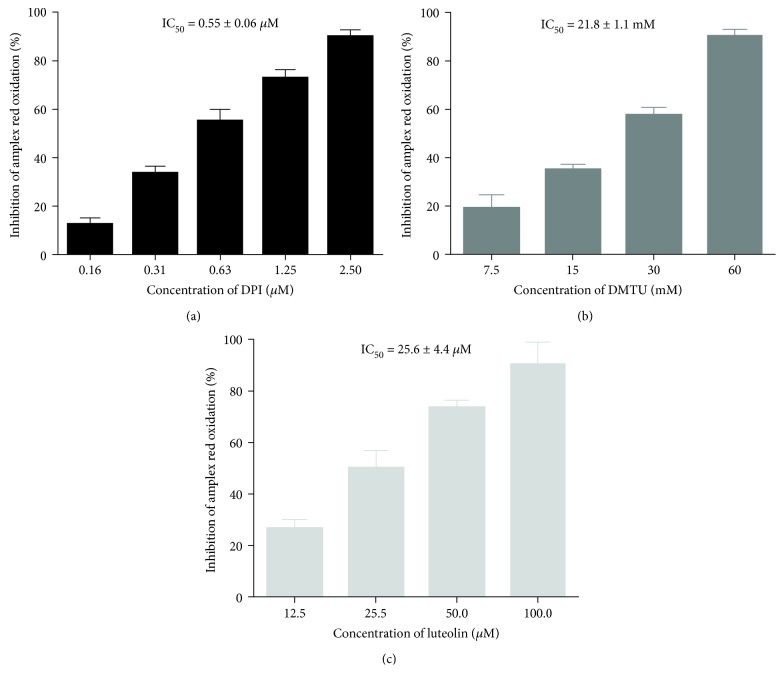
Inhibitory effects of DPI (0.16-2.5 *μ*M) (a), DMTU (7.5-60.0 mM) (b), and luteolin (12.5-100 *μ*M) (c) on the oxidation of amplex red, by whole blood-generated ROS, when stimulated by PMA. Values are given as mean ± SEM (*n* ≥ 3).

To guarantee that, in this model, amplex red continues to be a specific probe to detect H_2_O_2_, we tested the enzymatic inhibitors DPI (inhibitor of NADPH oxidase) and ABAH (inhibitor of MPO).  Figure 4 shows that 2.5 *μ*M of DPI totally hindered the oxidation of amplex red, demonstrating that the detection of H_2_O_2_ by amplex red is dependent on the NADPH oxidase activation. The lack of inhibition of amplex red oxidation by ABAH (data not shown) corroborates that HOCl production was not detected by this probe, in this cellular model. Catalase interferes with this method by inducing an HRP-independent oxidation of amplex red in the presence or absence of blood. This interference with the probe led us to use a scavenger of H_2_O_2_, DMTU, which inhibited the oxidation of amplex red in a concentration-dependent manner, proving that amplex red mainly detects H_2_O_2_. In addition, we also studied the flavonoid luteolin that was very effective in inhibiting the H_2_O_2_ production, presenting an IC_50_ of 25.6 ± 4.4 *μ*M.

### 3.3. Optimization of Experimental Settings in the APF Assay

The examination of the model regression coefficients (*P* < 0.05) showed that the qualitative factor (blood dilution) was not significant for the model and that the oxidation increases with the increase of APF and PMA concentration. The model for the percentage of APF oxidation [equation (3)] fitted the experimental data with a R^2^ of 0.98 and a Q^2^ value of 0.95, demonstrating that a model with a good fit and good predictability ability was obtained. In this case, a logarithmic transformation was made to the data to improve the model robustness.

(3) $$\begin{array}{c} \log_{10}\left(Oxidation\text{ of }APF\left(\%\right)\right)=1.26+0.289X_{1}+0.001X_{2}–0.018{X_{1}}^{2}. \end{array}$$

**Figure 5:**
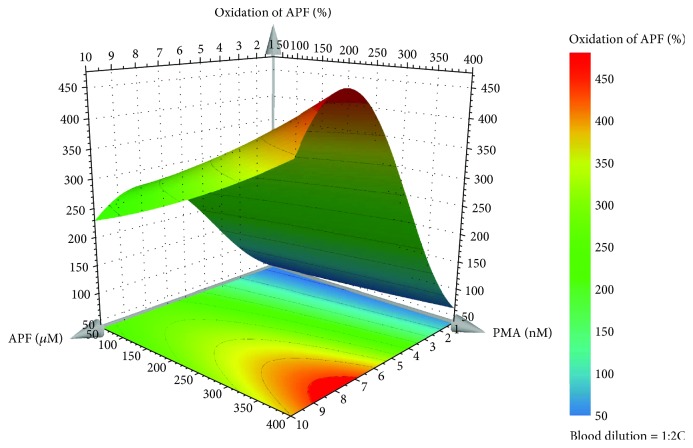
Response surface plot obtained for the percentage of oxidation of APF by the predictive model of the D-optimal design for the 1 : 20 blood dilution.

In equation (3), *X*_1_ is the APF concentration (*μ*M) and *X*_2_ is the PMA concentration (nM). Taking into account that all the tested dilutions of human blood originated a good range of oxidation percentage of the probe, we choose the 1 : 20 dilution in order to use less quantity of human blood in each assay, making the biologic sample more profitable. Once again, we choose 250% of the probe oxidation as an indicative value to validate the Equation. According to equation (3) and as it can be seen in the response surface plot (Figure 5), to obtain a percentage of oxidation of the probe around 250%, a concentration of APF and PMA of 5.5 *μ*M and 150 nM, respectively, was chosen. There were no significant differences (*P* < 0.05) between the validation experiments and those predicted by the model, confirming its good prediction ability. The validation of the model was executed (*n* = 6), and the results showed that the percentage of oxidation of the probe was around the expected value (257 ± 45%).

**Figure 6:**
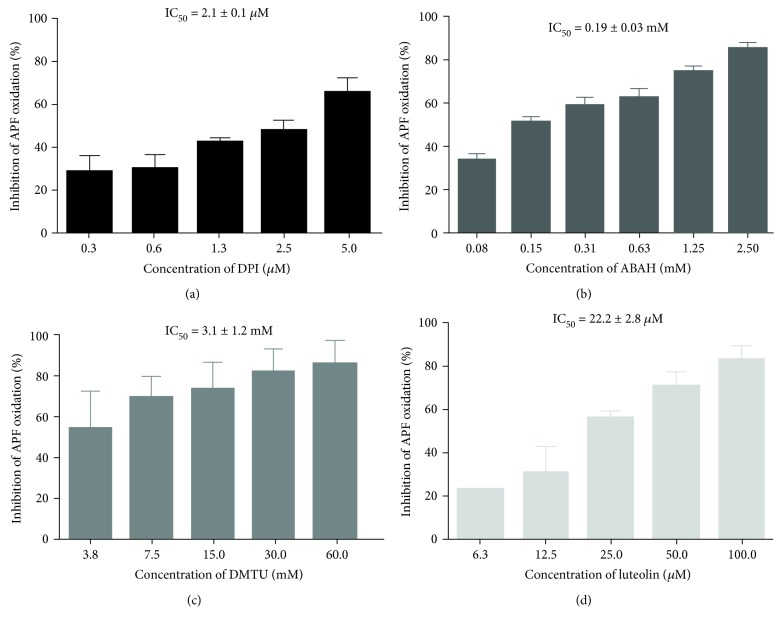
Inhibitory effects of DPI (0.31-5.00 *μ*M) (a), ABAH (0.08-2.50 mM) (b), DMTU (3.75-60.0 mM) (c), and luteolin (6.25-100 *μ*M) (d) on the oxidation of APF by whole blood-generated reactive species, when stimulated by PMA. Values are given as mean ± SEM (*n* ≥ 3).

As it can be seen in Figure 6, DPI, ABAH and DMTU almost avoided the oxidation of APF at the concentrations of 5.0 *μ*M, 2.5 mM, and 60 mM, respectively. Since DMTU scavenges H_2_O_2_, this could indicate that APF can detect H_2_O_2_ or other ROS that derived from H_2_O_2_, as HOCl. To test this cellular model, we also studied the flavonoid luteolin that was very effective in inhibiting the ROS production, presenting an IC_50_ = 22.2 ± 2.8 *μ*M.

## 4. Discussion

The detection of reactive species has been a matter of intense debate. Most of the studies described the detection of ROS in different cells types, primary or cell line, as neutrophils [14, 22], monocytes [23, 24], and macrophages [25]. However, in these studies lack the interaction among all the cells present in the blood, which could interfere and dictate a different behavior than that obtained using a single cell type. Therefore, here, we used human blood, from healthy donors, as cellular model, since it is a more complex and physiologic *in vitro* system, preserving all cell-cell and cell-matrix interactions. Besides that, the manipulation of this cellular model is easier, faster, and cheaper than the isolation process and the maintenance of a cell culture of a single cell type.

A D-optimal experimental design was used, for the first time, to optimize the experimental conditions for the *in vitro* detection of ROS produced by PMA-stimulated human blood cells, using fluorescent probes. The use of the D-optimal experimental design allows the optimization of the experimental conditions in a single step using few experiments. In this way, the region of interest is covered optimally by the chosen experimental settings. Moreover, this optimization and the provided equations increase the time and cost effectiveness of the experiments. For that purpose, three different but complementary fluorescent probes were used for ROS detection, namely, DCFH-DA, amplex red, and APF. The detection of reactive species was done using a common equipment of microanalysis, a microplate reader, which is cheaper and easy to use than other equipments used in this type of assays, as flow cytometer [26, 27] or HPLC [28]. In the DCFH-DA system, a quadratic equation was obtained with PMA concentration interacting with itself. This means that there is a quadratic relationship between the percentage of oxidation and the PMA concentration. The APF system also has a quadratic relationship between the response and the concentration of PMA. Moreover, in this system, the response was logarithmized to normalize the distribution of the response in order to improve estimates and statistics.

DCFH-DA has been used for many years for the detection of ROS in isolated cells such as leukocytes [3, 29, 30]. However, to the best of our knowledge, there are only two reports using DCFH-DA to detect ROS in human blood [3, 31]. Here, we innovate and optimized the experimental conditions using a D-optimal experimental design in order to achieve the conditions that best fit the objectives. Analyzing the response surface plot, and to obtain a percentage of oxidation of the probe of approximately 250%, it was possible to fix the concentrations of DCFH-DA and PMA into 120 *μ*M and 120 nM, respectively, and the blood dilution in 1 : 20.

DCFH-DA is a small, nonpolar, and nonfluorescent molecule that can diffuse into the cell, where intracellular esterases hydrolyze the acetate groups resulting in dichlorofluorescin that then reacts with a variety of ROS such as H_2_O_2_, HO^·^, and ROO^·^ and also with reactive nitrogen species such as ^·^NO, ^·^NO_2_, and ONOO^−^ [20], resulting in an increase in the fluorescent signal. Nevertheless, there is no information about which ROS are detected by this probe using a complex matrix as human blood. To clarify this point, we used the inhibitors of the most important enzymes responsible for the ROS production, such as DPI (inhibitor of several flavoenzymes, including NADPH oxidase [32]) and ABAH (inhibitor of MPO [33]). DPI and ABAH avoided the oxidation of DFCH-DA in a concentration-dependent manner. These results indicate that DCFH-DA can detect several ROS as O_2_^·-^ or H_2_O_2_ and also ROS derived from MPO, as HOCl. To understand which ROS, O_2_^·-^ or H_2_O_2_, were detected by DCFH-DA, we also tested catalase (catalyzes H_2_O_2_ decomposition into H_2_O) and DMTU (a cell-permeable scavenger of H_2_O_2_ [34]). Surprisingly, catalase increased the fluorescence of the probe by itself. As reported before, catalase may act as an intracellular factor able to oxidize fluorescent probes and also act as a cofactor for the reaction with H_2_O_2_, due to peroxidase activity [35]. Therefore, we used DMTU, which decreased the fluorescent signal, indicating that DCFH-DA essentially detects H_2_O_2_.

One of the main advantages of DCFH-DA assay is its simplicity of use, as it can be seen in the materials and methods section, but it also has other advantages as high sensitivity, an affordable price, and a nonselective detection of intracellular ROS, providing an overview of the overall prooxidant status. Nevertheless, the use of this probe has also some limitations that should be taken into account. One of them is the autooxidation and photoreduction, whether in the presence of visible light or by action of UVA radiation, that can be easily overcome by properly protecting the probe [20, 36]. The presence of antioxidants, naturally present in the cells or in the culture media, could compete with the probe for reaction with ROS resulting in the underestimation of ROS production. As such, it is important to use a high probe concentration to establish appropriate intracellular concentration or stimulate the cells properly to overcome this interference [36].

We also tested amplex red, which selectively reacts with H_2_O_2_ at the stoichiometry of 1 : 1 in a reaction catalyzed by HRP to generate the highly fluorescent product resorufin [37]. H_2_O_2_ is one of the most stable forms of ROS; thus, its detection allows the observation of oxidation processes in real time. Similar to DCFH-DA, amplex red has been used in isolated leukocytes [29, 38]. There is only one paper describing the use of amplex red in rat blood applied for the measurement of acetylcholinesterase activity [39]. The use of amplex red as probe for the detection of H_2_O_2_ in human blood is described here for the first time.

Interestingly, our results show that the percentage of amplex red oxidation increases with the decrease of amplex red concentration, indicating a higher sensitivity for lower concentrations of amplex red. This could be related with the higher baseline background fluorescence caused by higher concentrations of amplex red. This effect was already reported by Mohanty et al. [40], which stated that reducing the concentration of amplex red from 50 *μ*M to 10 *μ*M, increased the detection limiting, enabling the detection of 2 pmol instead of 0.1 to 2 nmol of H_2_O_2._ Accordingly, by analyzing the response surface plot derived from a D-optimal experimental design, the concentration of amplex red was fixed at 10 *μ*M together with 150 nM of PMA and the blood dilution 1 : 20 to obtain a percentage of oxidation of the probe around 250%. DPI totally inhibited the oxidation of amplex red, showing that it detects H_2_O_2_ that can derive from NADPH oxidase and/or mitochondria activities. Catalase and DMTU were also tested. Once again, catalase interfered with the assay, increasing by itself, the fluorescent signal, independently of the presence of blood and PMA in the reaction mixture. The depletion of fluorescent signal induced by DMTU, together with the absence of an effect of ABAH, reinforces the idea that amplex red selectively detects H_2_O_2_. As such, using blood as cellular model, amplex red continues to be highly sensitive and selective for H_2_O_2_ detection. In addition, resofurin, the highly fluorescent product of amplex red oxidation, is stable, and its longwave spectrum avoids inference from autofluorescence originated in biological samples [37]. It is important to note that this methodology, as other HRP-dependent methodologies, is susceptible to the interference from substances that oxidize this enzyme [1]. Nevertheless, given its high sensitivity, specificity, and chemical stability, amplex red is suitable for the detection of H_2_O_2_ in human blood.

APF was successfully used to quantify MPO activity in isolated cells by few researchers [14, 15]. To the best of our knowledge, there is only one report from our group, using APF in human blood [31]. Once again, we felt that the literature lacks information about this issue, and most importantly, the optimization process should be clarified. As it was mentioned above, there is a current need to use human blood as a cellular model to truthfully understand the influence of all blood components in the activity of anti- or prooxidant compounds. In the case of APF, we have to perform a hypotonic lysis of erythrocytes before the reading of the fluorescent signal in the microplate reader. This step was essential to obtain a difference of values between the blank (without PMA) and the control (with PMA) assays. Our procedure is in agreement with Flemmig et al. [41], who used a flow cytometer to detect the APF fluorescence in human blood and also reported the elimination of erythrocytes. However, in contrast with what was reported in the work of Flemmig et al. [41], in the present study, the blood was incubated with PMA and/or the compounds under study, before the erythrocytes lysis, precisely to guarantee their interaction with the different type of the cells, including erythrocytes. In addition, here, we proposed an analysis in a microplate reader that is of easy use and cheaper than a flow cytometer.

APF is a nonfluorescent derivative of fluorescein that detects HOCl, HO^·^, and ONOO^−^ intracellularly [42]. Our previous reports have shown that APF is more sensitive to HOCl than to the other ROS [14, 15, 43]. As it was done with the other probes, we also tested several inhibitors of the ROS production. ABAH also avoided the production of reactive species, suggesting that HOCl strongly contributes to APF oxidation. Interestingly, DMTU also inhibited the APF fluorescent signal. This indicates that H_2_O_2_ influences, directly or indirectly, the oxidation of APF. As the majority of the produced H_2_O_2_ is used by MPO to originate HOCl, it seems that DMTU removes the produced H_2_O_2_ that will not be consumed by MPO and consequently originates a decrease in the fluorescent signal.

Compared to the other probes, APF is not light sensitive; however, it is the more expensive and time-consuming probe [42]. Nevertheless, APF allowed the selective detection of one of the main ROS produced during the inflammatory process. The overproduction of HOCl is related to the development of several human diseases such as arthritis, cancer, and neurodegeneration [44]. As such, due to its biological importance, the use of sensitive and selective probes for its detection is of utmost importance.

To validate the methods discussed here, luteolin, a known antioxidant, was used as a positive control. Luteolin decreased the production of ROS, using all of the three probes, proving its ability to modulate the production of reactive species, also in a complex cellular model, as human blood, as it was already described in other type of *in vitro* assays, using isolated human neutrophils [14–16].

## 5. Conclusions

In this work, a D-optimal experimental design was used for the first time to optimize the experimental conditions for the *in vitro* detection of ROS produced by human blood, from healthy donors, using three different and complementary fluorescent probes, DCFH-DA, amplex red, and APF. Our results will help researchers to accurately choose the conditions that better fit their scientific objectives, saving time and money, and most importantly, using a physiological *in vitro* system that mimics the *in vivo* settings and which is yet unexplored.

## Data Availability

The data used to support the findings of this study are included within the article.
